# RNA and Proteins: Mutual Respect

**DOI:** 10.12688/f1000research.10572.1

**Published:** 2017-03-27

**Authors:** Kathleen B. Hall

**Affiliations:** 1 Department of Biochemistry and Molecular Biophysics, Washington University School of Medicine, St Louis, MO, 63110, USA

**Keywords:** Ribonucleoprotein Particles, RNPs, RNA-protein interaction, Xist, lncRNA, U4/5/6 tri-snRNP complex

## Abstract

Proteins and RNA are often found in ribonucleoprotein particles (RNPs), where they function in cellular processes to synthesize proteins (the ribosome), chemically modify RNAs (small nucleolar RNPs), splice pre-mRNAs (the spliceosome), and, on a larger scale, sequester RNAs, degrade them, or process them (P bodies, Cajal bodies, and nucleoli). Each RNA–protein interaction is a story in itself, as both molecules can change conformation, compete for binding sites, and regulate cellular functions. Recent studies of Xist long non-coding RNP, the U4/5/6 tri-small nuclear RNP complex, and an activated state of a spliceosome reveal new features of RNA interactions with proteins, and, although their stories are incomplete, they are already fascinating.

## Introduction

RNA molecules in the cell are rarely naked. Rather, proteins are bound to them in some arrangement consistent with their regulation, protection from nucleases, transport, or formation of ribonucleoprotein particles (RNPs). A 2014 compendium of RNA-binding proteins in humans ^1^ concluded that 7.5% of 20,500 known protein-coding genes are found in RNPs or bound to mRNAs, where they regulate RNA metabolism. This is likely to be an underestimate, since their structural heterogeneity makes them difficult to identify *de novo*.

The recent discovery of a plethora of non-coding RNAs ^2^ in cells has invigorated investigation of proteins that bind to RNA. New methods of probing the proteins in a transcriptome have allowed simultaneous identification of a protein and its RNA-binding site. Typically, these are crosslinking-immunoprecipitation (CLIP) experiments ^3– 9^. Intact cells can be irradiated with ultraviolet (UV) light or treated with formaldehyde to crosslink proteins to RNA, then the complexes are purified from the milieu by immunoprecipitation. To identify proteins bound to mRNAs, cellular UV RNA–protein crosslinking is followed by isolation of all poly(A)-RNA ^7^. Alternatively, proteins bound to a specific RNA could be recovered by annealing biotin-oligonucleotides complementary to the RNA and selective purification by streptavidin ^9^. Proteins bound to RNAs could then be identified by mass spectrometry. Several groups applied this method to identify mRNA-binding proteins in human cell lines, mouse embryonic stem cells (ESCs), and *Saccharomyces cerevisiae* yeast cells (reviewed in Gerstberger *et al*. ^1^).

Assuming that there are indeed more than 1,500 RNA-binding proteins in human cells, books will be written about them and their roles in RNA biology. Here, I focus on recent advances that reveal the variety and mystery of RNPs.

## Xist, the RNA that inactivates an X chromosome

**Figure 1.**
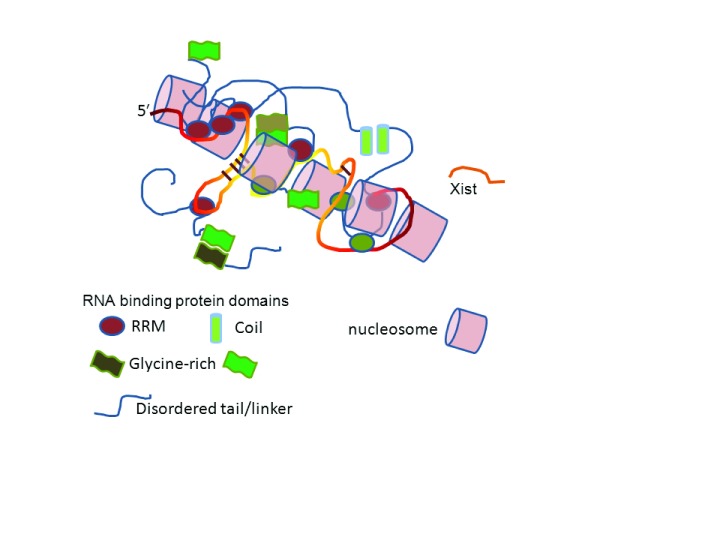
Xist wraps around nucleosomes in the X chromosome. Approximately 200 Xist molecules bind to an X chromosome, spread along it, and inhibit RNA polymerase II from transcribing the DNA. Xist is bound by many proteins at unknown sites and with unknown stoichiometry, which subsequently interact with each other through disordered regions or structured domains. RNA is shown as a yellow/orange strand and protein linkers as blue strands. RRM, RNA recognition motif.

Xist is a long non-coding RNA (lncRNA) that is responsible for transcriptional silencing of one of two X chromosomes in female cells ^10– 13^. There are approximately 200 Xist molecules bound to a single X chromosome, and each 18 kb of Xist is bound by proteins ( Figure 1). Proteins could participate in any aspect of its biology: Xist has to associate with the X chromosome, then spread along it, and finally inhibit RNA polymerase II (Pol II) transcription. After more than twenty years of efforts to identify those proteins, the power of mass spectroscopy has been applied to proteins crosslinked *in cellulo* to Xist.

Two research groups have recently published compendia of Xist-bound proteins. Each group first crosslinked RNA to protein *in cellulo*, selected Xist through oligonucleotide-directed annealing, then used quantitative mass spectrometry to identify bound proteins. An overall comparison of their results shows great similarity but also some curious and intriguing differences. Table 1 and Table 2 list the most abundant proteins recovered from each study.

**Table 1.** Top 15 Xist-binding proteins from Chl-MS recovery in mouse cells ^14^.

Crosslinked proteins	In order of abundance	Protein structural motifs	Length * (number of amino acids)
hnRNP M	1	3 RRM	728
hnRNP U (Saf-A)	2	RGG, KH, acidic region, DNA binding	793
hnRNP K	3	3 KH, proline-rich	463
hnRNP A2/B1	4	2 RRM, RGG, glycine-rich	353
MYEF2	5	2 non-canonical RRMs, homology to hnRNP M4	591
hnRNP A1	6	2 RRM, glycine-rich, RGG	320
DDX5	7	DEAD box protein	
Spen (SHARP)	8	3 RRM, SPOC	3,640
RBM XL1	9	RRM	
hnRNP AB	10	2 RRM	
hnRNP D (AUF1)	11	2 RRM	355
hnRNP L	12	4 RRM, glycine-rich	589
hnRNP A3	13	2 RRM, glycine-rich	379
hnRNP C	14	1 RRM, acid rich	293
TARDBP (TDP-43)	15	2 RRM, glycine-rich, DNA-binding protein	414

*Many proteins have isoforms with varying lengths; the longest variant in *Homo sapiens* is listed.

**Table 2.** Mouse embryonic stem cells: top 10 Xist-binding proteins from RAP-MS ^23^.

Crosslinked proteins	In order of abundance	Protein structural motifs	Length (number of amino acids)
SHARP (SPEN)	1	3 RRM, SPOC	3,640
RBM15	2	3 RRM, SPOC	969
MYEF2 (hnRNP M)	3	3 RRMs, homology to hnRNP M4	591
CELF1	4	3 RRMs	486
hnRNP C	5	1 RRM	313
LBR	6	Chromatin-interaction domain, transmembrane region, lamin-interacting domain	626
SAF-A (hnRNP U)	7	RGG, SPRY domain, ATPase domain	793
RALY (hnRNP C)	8	1 RRM	312
hnRNP M	9	3 RRM	729
PTBP1 (hnRNP I)	10	4 RRM	555

hnRNP, heterogeneous nuclear ribonucleoprotein particle; RAP-MS, RNA antisense purification-mass spectrometry; RRM, RNA recognition motif; SILAC, stable isotope labeling by amino acids in culture; SPOC, Spen paralog and ortholog C-terminal domain.

The groups of Heard and Chang ^14^ identified 81 proteins *in toto* bound to Xist. Using formaldehyde, they crosslinked proteins to Xist in three different mouse cell types: a male ESC line containing an inducible Xist gene, an epiblast stem cell line, and trophoblast stem cells. Each cell type represents one stage of Xist expression. Combining all datasets, three proteins were identified as being most abundant: heterogeneous nuclear RNP (hnRNP) K, hnRNP U, and hnRNP M. In addition, a detailed examination of Xist 5′ 0.9 kb sequence revealed several localized proteins. In particular, SPEN (aka SHARP) was found to be necessary for transcriptional silencing.

There is a preponderance of hnRNP proteins. These heterogeneous nuclear ribonucleoproteins are abundant in metazoan cells, where they are mostly found in the nucleus ^15, 16^. A recent review of them traced their ancestry ^17^, concluding that there are 13 families, each with isoforms or variants. For example, hnRNP A has four homologues in humans (A0, A1, A2, and A3), while hnRNP M has two (MYEF2 and hnRNP M). These proteins typically use RNA recognition motifs (RRMs) to bind RNA, while their other domains engage in protein–protein interactions. Several are involved in pre-mRNA splicing, where they repress splice site selection (hnRNP A ^18^) or regulate exon inclusion (hnRNP I ^19^). hnRNP I (aka polypyrimidine tract binding protein 1 [PTB1]) also facilitates translation from internal ribosome entry sites (IRES) ^20, 21^. hnRNP functions in Xist are unknown, with the exception of hnRNP U (aka Saf-A), which facilitates Xist localization on chromatin ^22^.

In contrast, a group of investigators headed by Guttman ^23^ took a different approach to finding Xist proteins during transcriptional silencing. After Xist induction in mouse ESCs, cells were UV-crosslinked, Xist RNP was recovered with long antisense oligonucleotides, and Xist proteins were identified by mass spectrometry. Two batches of mouse ESCs were cultured, one in ^15^N- and one in ^14^N-media to allow quantification by mass spectrometry (SILAC). Among their ten most abundant proteins, they found SHARP (SPEN) and RMD15, two proteins related in their architecture (they are SPEN family proteins). They also recovered six hnRNP proteins ( Table 2).These are exciting findings. In a curious coincidence, SHARP has another life in a nuclear RNP with the steroid receptor RNA activator (SRA) ^24^. SRA is a lncRNA that co-regulates the transcription of nuclear receptors ^24^. Bound to SRA, SHARP represses SRA transactivation when it recruits histone deacetylate ^25^. Does it carry out a similar task in Xist ^2, 10^?

In fact, McHugh *et al*. found that SHARP was required for the inhibition of Pol II transcription at sites where Xist was bound ^23^. The mechanism of inhibition could lie in the recruitment of SMRT and/or HDAC3 ^25^. HDAC3 is a histone deacetylase ^26^ that is thought to be responsible for transcriptional repression by changing chromatin structure ^27^. Loss of SHARP, LBR, or hnRNP U in knockdown experiments was sufficient to eliminate silencing ^23^, but each protein appears to have unique contributions. The role of the other seven proteins was not tested directly, but since each binds directly to Xist, they could have functions in localization, recruitment of other enzymes, stabilization, etc. (for example, binding to Polycomb repressive complex 2 [PRC2]).

The identification of LBR bound to Xist explains localization of the Xist-X chromosome to the nuclear lamina ^12^. Transmembrane helices anchor LBR to the lamina, while its tail contacts Xist. Positioning of Xist-X on the lamina changes the structure of the DNA and facilitates protein-mediated spreading of the Xist molecules along the length of the chromatin.

Rather than discovering unknown proteins, these investigations have re-discovered known proteins. They present a new challenge: to understand why they are particularly useful in the Xist context and how their use, and corresponding abundance, is modulated according to developmental stage or cell lineage. The general challenge is not only to understand how proteins use their RNA-binding domains and intervening sequences and disordered tails to control formation of RNPs but must also account for their temporal exchange.

## RNA recognition motifs

A striking feature of proteins bound to Xist is the recurring use of tandem RRM domains. There are certainly advantages to this scheme, since affinity and specificity can be modulated by increasing the number of contacts between RNA and protein. However, neither Xist-binding sites for its associated proteins nor their binding stoichiometry are known. These biochemical characterizations are important to understand how they select their target sites on the RNA, how they bind to Xist in the milieu of other RNAs in the cell, and how they hang onto the RNA while they also bind to other cellular compartments or recruit other proteins.

**Figure 2.**
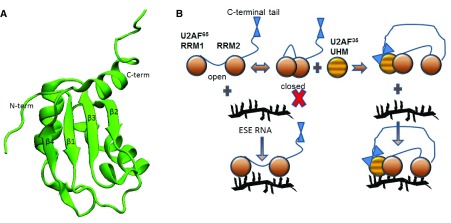
Regulation of RNA recognition motif (RRM) binding to RNA. **A**. An RRM has a four-stranded anti-parallel β-sheet, with two α-helices on one side ^92– 95^. RNA often sits on the surface of the β-sheet. **B**. The two RRMs of U2 auxiliary factor (U2AF) exhibit closed/open transitions at equilibrium, but only in the open state can RNA bind. Binding of the U2AF homology motif (UHM) from U2AF35 to RRM1 shifts the equilibrium to favor the open state, which facilitates RNA binding. The C-terminal tail of U2AF65 contacts the U2AF35 UHM. ESE, exonic splicing enhancer.

RRMs ^1^ are the most common structural motif used in eukaryotes to bind RNA ( Figure 2) and are estimated to be found in 225 human genes. When RRMs are present in multiples, deciphering the contributions of each RRM to the whole can be quite difficult ^28– 31^. A recent biophysical study of two tandem RRMs revealed how they partition function.

U2 auxiliary factory (U2AF) is a heterodimer of U2AF65 and U2AF35 ^32, 33^, which in pre-mRNA splicing aids in the recognition of a 3′ splice site ^34– 38^. U2AF65 has two RRMs (RRM1 and RRM2) that bind polypyrimidine tracts, but U2AF35 has a single UHM, a “U2AF homology motif”, that is structurally homologous to an RRM ^39, 40^. RRM1 and RRM2 are tethered by a short linker (~20 amino acids) that allows them to undergo relative motion and orientation ^36^. Since they bind to polypyrimidine tracts of variable length and sequence, they must be able to expand or contract to span the site ^41^.

The Sattler and Lamb laboratories collaborated on a comprehensive study of the spatiotemporal disposition of U2AF65 RRM1 and RRM2 and their role in RNA binding. von Voithenberg *et al*. ^35^ showed that RRM1 and RRM2 undergo dynamic exchange between a closed or open orientation at equilibrium ( Figure 2). In the closed state, RRM1 and RRM2 do not bind RNA, but when the conformation is open, a polypyrimidine tract can bind. If binding is weak (i.e. the polypyrimidine tract is too short or contains multiple purine nucleotides), the exchange between open and closed states is relatively unperturbed. If RNA binding is tight, RRM1 and RRM2 will be trapped in an open state. Thus, the RNA shifts the equilibrium of U2AF RRM1 and RRM2 between open and closed states in an example of conformational selection.

These experiments were conducted using single pair Förster resonance energy transfer (spFRET) that observed single molecules, each containing a donor and acceptor fluorophore. One fluorophore was attached to either RRM, such that the open and closed orientations were distinguished by the FRET efficiency. Combining measurements of fluorophore lifetimes with spFRET facilitated temporal characterization of exchange between open and closed states. In experimental conditions, free RRM1 and RRM2 occupied an open state ~67% of the time. Addition of RNA trapped RRM1 and RRM2 in the open conformation 90% of the time.

U2AF65 and U2AF35 have been the subject of many biochemical and structural investigations, since they are essential proteins for pre-mRNA splicing. In particular, experimental studies of protein–protein interactions between U2AF and other proteins have identified sites where interactions occur ^42– 45^. These latest experiments revealed a mechanism of protein–protein interaction involving the UHM of U2AF35 and U2AF65 RRM1 and RRM2. A combination of nuclear magnetic resonance (NMR) structure and dynamics experiments identified the binding site of U2AF35 UHM to be a surface of U2AF65 RRM1. Binding of the UHM to RRM1 shifts the RRM1 and RRM2 conformational equilibrium to the open state, thereby favoring RNA binding. The authors suggest that allostery drives the RRM1 and RRM2 conformational switch. Allosteric modulation of binding is a powerful mechanism to provide discrimination and affinity ^46– 49^, but, by its nature, it is almost impossible to anticipate and cannot be gleaned from static structures.

Many RNA-binding proteins are modular, with an RNA-binding domain, intervening sequences, and disordered tails. Here, U2AF uses two proteins to regulate splicing; other examples include the Sxl-Unr heterodimer that regulates translation via interactions between Sxl RRM and a Unr cold-shock domain ^50^, while the SR protein (serine-arginine) SRSF1 is regulated by phosphorylation of its RS tail that blocks intramolecular interaction with its RRMs ^51, 52^. Regulation by intermolecular and intramolecular interactions adds another level of complexity to RNA-binding proteins.

## The spliceosome and its small nuclear ribonucleoprotein particles

It is estimated that 94% of all human genes contain introns ^53– 55^, thereby providing protein isoform diversity. The process of removing introns and joining exons is carried out by the spliceosome, a multi-component and dynamic assembly of RNPs ^56^. A great challenge in the field of pre-mRNA splicing has been to understand how the spliceosome is physically able to carry out the concerted transesterification reactions of the splicing chemistry to yield mRNAs.

The spliceosome consists of five small nuclear RNPs (snRNPs) that dynamically associate with each other and with pre-mRNA. The major spliceosome uses U1, U2, U4, U5, and U6 snRNPs in the process of splicing ^57^. Each snRNP contains a single RNA (snRNA) and multiple proteins, but while U1 and U2 snRNPs are independent, U4 and U6 form a di-snRNP that goes on to become a U4/U5/U6 tri-snRNP ^58^. The tri-snRNP is recruited to a bona-fide intron and is then remodeled, losing U4 snRNP and leaving U5 and U6 snRNPs to form the active spliceosome.

The goal of snRNP rearrangement is to allow and facilitate snRNA conformational rearrangements in the spliceosome to produce the active site for catalysis ^59– 61^. Rearrangements of pre-mRNA and snRNAs to prepare and position them for catalysis are mainly accomplished by protein helicases ^62^. There are eight such type SF2 helicases that associate with the spliceosome along the reaction pathway ^63, 64^. These ATP-dependent RNA helicases are not sequence specific; they can unwind any RNA duplex. Rather, their specific targets appear to be defined by where and when they associate with the spliceosome. The Brr2 helicase is particularly critical in the transformation of pre-spliceosome intermediates ^64– 67^. Brr2 is unusual: it has two helicase domains (only one is active) and a long (450-amino-acid) N-terminal domain ^64, 65, 68, 69^.

## Brr2, a unique RNA helicase

Brr2 enters the nucleus independently and associates with the U5 snRNP. U5 snRNP then joins the U4/U6 di-snRNP to become the U4/U5/U6 tri-snRNP ^68^. The tri-snRNP is recruited by U1 and U2 snRNPs to form a pre-spliceosome.

To form the active spliceosome, two snRNPs must be displaced. U1 snRNP is released from the 5′ splice site, and U4 snRNP is removed from the tri-snRNP. It is the latter remodeling that requires Brr2, as U4 and U6 snRNAs are joined by 22 perfect base pairs and Brr2 is the helicase that separates them. Only when U6 snRNA is free of U4 snRNA can it rearrange to base pair with U2 snRNA and pre-mRNA and so form the catalytic center of the spliceosome. Clearly, Brr2 activity must be regulated such that it is inactive in the tri-snRNP but active in the pre-spliceosome. How is it regulated?

Several recent studies have delved into the details of Brr2 regulation. In a series of papers from the Wahl lab ^70– 74^, Brr2 structure and function were addressed by crystallography and biochemistry. The goal of Brr2 in the tri-snRNP is to maintain stasis. As biochemistry experiments of Brr2 show ^64^, there is a plug domain at the N-terminus of Brr2’s long N-terminal region (NTR). This plug folds back over the entrance of the helicase to block access of the U4/U6 snRNA duplex to the active site of Brr2. This is a unique intramolecular regulatory device, and more experiments are required to understand how it is directed to this position (and how it is displaced).

**Figure 3.**
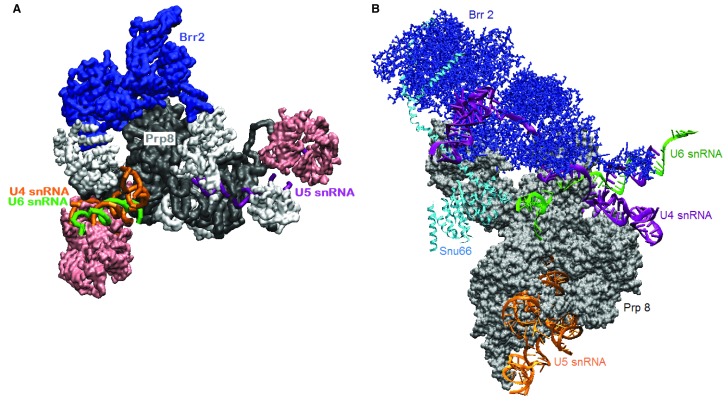
Two tri-small nuclear ribonucleoprotein particle (snRNP) structures trap different states of Brr2. **A**. Human tri-snRNP cryo-electron microscopy (cryo-EM) at 7 Å resolution ^72^ shows Brr2 sitting on Prp8 (PDB ID 3jcr). A U4/U6 snRNA duplex is visible. Sm and Lsm rings are pink; other proteins are white. **B**. In a yeast tri-snRNP complex ^70^, (PDB ID 5GAN), U4 snRNA is threaded through Brr2 in the RNA-binding tunnel. These structures might correspond to the tri-snRNP in the nucleus ( **A**) and the tri-snRNP poised for activation by Brr2 as it joins the pre-spliceosome ( **B**). Visualized with visual molecular dynamics (VMD).

The tri-snRNP is an intermediate in the pathway to spliceosome formation. Years of enormous efforts to map intermediates ^42, 63, 75– 77^ have now been coupled with technological advances in cryo-electron microscopy (cryo-EM) to visualize select transitional complexes ^70– 72, 78, 79^. Those efforts have produced a cryo-EM structure of human tri-snRNP that captures Brr2 in its plugged conformation ^72^ (PDB ID 3jcr). This state of the tri-snRNP, illustrated in Figure 3, might represent its structure as an autonomous particle before it joins the pre-spliceosome, where U4 and U6 snRNAs are still base-paired to each other. If so, then proteins and RNAs in the tri-snRNP must rearrange to present U4 and/or U6 tails to the helicase active site.

In the tri-snRNP, Brr2 sits on the Jab1 domain of Prp8, but its orientation and contacts change during activation of the particle. In contrast to the structure of the human tri-snRNP, in a structure of yeast tri-snRNP, a single-stranded region of U4 snRNA occupies the RNA-binding tunnel of Brr2 ^73, 80, 81^ (illustrated in Figure 3). Is Brr2 now poised to completely separate U4 snRNA from U6 snRNA? Does this separation occur before the tri-snRNP is recruited to the pre-spliceosome, or is this a paused state that requires further activation?

There is another competitive inhibitor of Brr2. Prp8’s Jab1 domain has a C-terminal disordered tail that sneaks into the RNA tunnel of Brr2 to compete with U4 ^82^. The intramolecular plug interaction and Prp8 Jab1 cooperate to inhibit unwinding. Removing the Jab1 tail activates Brr2 helicase activity; Brr2 without its intramolecular plug also has enhanced activity ^75^. Do both inhibitors operate in the isolated tri-snRNP?

**Figure 4.**
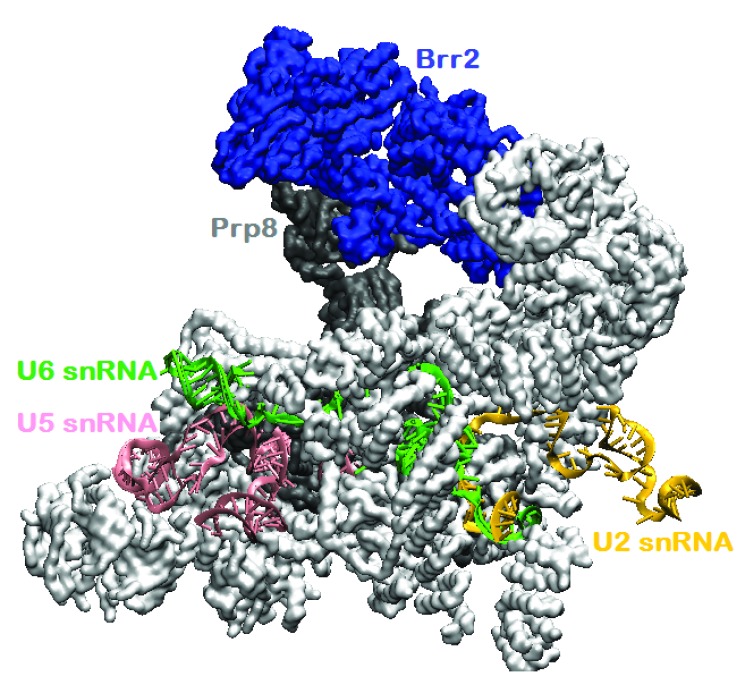
Yeast-activated (B ^act^) spliceosome ^79^ (PDB ID 5LQW; cryo-electron microscopy [cryo-EM] 5.8Å). Brr2 has separated U4 and U6 small nuclear RNAs (snRNAs), and U4 small nuclear ribonucleoprotein particle (snRNP) has been expelled from the spliceosome. Brr2 is bound to the Jab1 domain of Prp8. All 27 proteins are shown in surface representation; most are colored white. Visualized with visual molecular dynamics (VMD).

Brr2 remains in the spliceosome after U4 snRNP has been expelled from the spliceosome. It is seen in a structure of yeast-activated spliceosome, which is defined by the loss of U1 and U4 snRNP and rearrangements of the remaining snRNAs to interact with each other and pre-mRNA. A cryo-EM structure of activated yeast spliceosomes (B ^act^) shows Brr2 perched on Prp8’s Jab1 domain ^79^, with its helicase activity blocked by both inhibitor interactions (PDB ID 5lqw). In an illustration from this structure, U2, U5, and U6 snRNAs are remote from Brr2 ( Figure 4). Although not clear from the perspective of Figure 4, Prp8 is entwined with other proteins and the snRNAs in this complex, even as it binds Brr2.

As the spliceosome progresses through its cycle, there are many short RNA duplexes that need to be unwound. The other seven SF2 RNA helicases are recruited to the spliceosome when they are needed, and then they dissociate. Brr2 remains with the spliceosome until it has completed a splicing cycle, but there are no data suggesting that it is active at any time other than in the conversion from pre-spliceosome to B ^act^. If it is not required for its helicase activity, perhaps its long NTR contributes something to splicing. Brr2 is reported to contribute to catalysis ^74, 83^, to stabilize U5 and U6 in the spliceosome ^68^, and to assist in the final disruption of the spliceosome and release of ligated exons ^84^. If these states of the spliceosome could be trapped for structural studies, Brr2 might be captured in action.

The spliceosome is composed of hundreds of proteins ^56^, many of which simply bind RNA, but others actively remodel it. In the past year, spliceosome structures have revealed connections between RNA and proteins that explain previous observations but also raise new questions. This year, structures of the spliceosome C/C* complex show another helicase, prp16, at work on remodelling ^85– 87^. Slowly, this RNA enzyme is giving up its secrets.

## Conclusions

There is a need to not only understand specific RNPs but also define general rules of engagement, since RNA–protein interactions dominate RNA biology. Indeed, the most mysterious are the membrane-less organelles that contain RNAs and proteins ^88, 89^. These conglomerates of RNAs bound by RNA-binding proteins are variously thought to be centers of RNA processing, degradation, transcription, and exchange: P bodies and stress granules in the cytoplasm and nucleoli, Cajal bodies, speckles, and PML bodies in the nucleus. A current model is that disordered domains of the proteins form a fluid matrix that allows a flux of molecules through these liquid droplets ^90, 91^. It is a sure bet that these droplets will be objects of intense scrutiny for years to come.
